# Epithelial–Mesenchymal Transition Increases the Susceptibility of Human A549 Cells to Nanosecond Pulsed Electric Fields

**DOI:** 10.3390/ijms262311360

**Published:** 2025-11-24

**Authors:** Manato Mitsui, Keiko Morotomi-Yano, Ken-ichi Yano

**Affiliations:** 1 Faculty of Advanced Science and Technology, Kumamoto University, Kumamoto 860-8555, Japan; 2 Institute of Industrial Nanomaterials, Kumamoto University, Kumamoto 860-8555, Japan

**Keywords:** epithelial–mesenchymal transition, nanosecond pulsed electric field, cell death, nanopore

## Abstract

Nanosecond pulsed electric fields (nsPEFs) are ultrashort, high-intensity electrical pulses that have unique biological actions, including the formation of membrane nanopores and the efficient induction of cell death. nsPEFs are currently regarded as a promising modality for cancer therapy. During cancer progression, abnormal cells become more malignant through epithelial–mesenchymal transition (EMT). EMT is a biological process in which cells acquire mesenchymal traits and thereby increase their motility and invasiveness. Despite the importance of EMT in cancer progression, however, limited information is available on whether EMT modulates cellular responses to nsPEFs. In this study, we compared the responses of EMT and non-EMT human A549 cells to nsPEFs. We found that EMT induction rendered A549 cells more susceptible to nsPEFs as evidenced by decreased cell viability and increased nanopore formation upon nsPEF exposure. nsPEFs predominantly induced non-apoptotic cell death in EMT cells, and extracellular Ca^2+^ augmented the cytotoxicity of nsPEFs, suggesting involvement of nanopore-mediated Ca^2+^ influx in cytotoxicity. These findings reveal a novel feature of nsPEFs in targeting EMT cells, further supporting the unique biological actions of nsPEFs and their therapeutic potential for cancer.

## 1. Introduction

Nanosecond pulsed electric fields (nsPEFs) are considered a promising physical method for various applications in biology and medicine [1,2,3]. To date, the biological actions of nsPEFs have been extensively investigated using cultured cell lines and animal models. When applied to cells, nsPEFs elicit various cellular responses. First, nsPEFs induce the formation of small membrane pores, called nanopores, on the surface [4,5,6]. Nanopores allow the permeation of ions and small compounds, and their formation can be visualized by the intracellular incorporation of YO-PRO-1, a membrane-impermeant dye [4,6]. Nanopore formation disrupts the ionic balance across the cell membrane, such as inducing Ca^2+^ influx, which compromises cellular homeostasis [7,8,9,10,11]. Exposure to nsPEFs also causes impairment of mitochondrial activity and the consequent onset of metabolic dysfunction [12,13,14]. Furthermore, nsPEF exposure activates various intracellular signaling pathways, including stress responses [15,16,17]. Collectively, nsPEFs have unique and profound effects on the cell surface and intracellular components.

A prominent feature of nsPEFs is their remarkable capability to elicit cell death. nsPEFs appear to exert cytotoxicity through the combined effects on the cell surface and intracellular components. Previous studies have shown that nsPEFs trigger either apoptosis or non-apoptotic cell death depending on the cell type and experimental conditions [18,19,20,21,22]. In addition, cells exposed to nsPEFs frequently exhibit the hallmarks of immunogenic cell death [23,24,25]. In line with their high cytotoxicity and immunogenic effects at the cellular level, multiple animal studies have demonstrated that nsPEFs are highly effective not only for tumor ablation but also for preventing tumor recurrence [24,26,27]. Accumulating evidence from both in vitro and animal studies highlights the therapeutic potential of nsPEFs, and their use is currently explored in clinical trials [28,29].

Despite substantial scientific and therapeutic efforts, cancer remains one of the major causes of human mortality across the world [30]. Cancer development begins with the rapid, uncontrolled proliferation of abnormal cells, which subsequently gives rise to a primary tumor [31,32]. In general, the primary tumor itself is not the direct cause of death, but it often provides a microenvironment in which a subset of cells can acquire increased motility and invasiveness, ultimately becoming metastatic [31,32]. Metastatic cells disseminate to distant organs and compromise various normal physiological functions, ultimately leading to death. Cancer cells acquire metastatic properties through a biological process termed epithelial–mesenchymal transition (EMT). EMT is a dynamic cellular program in which epithelial cells lose their characteristic polarity and adhesiveness while acquiring the motile and invasive traits typical of mesenchymal cells [33,34,35].

EMT is driven by extensive transcriptional reprogramming that leads to broad molecular and structural alterations. First, EMT involves the reorganization of membrane proteins [35,36,37]. Epithelial genes, such as E-cadherin and cytokeratins, are downregulated, while mesenchymal genes, including fibronectin and vimentin, are upregulated [36,37]. These changes in the expression patterns of membrane proteins reduce intercellular adhesiveness and promote cell detachment. Concurrently, a significant fraction of actin filaments is reorganized from the actin cortex lying beneath the cell membrane into stress fibers and lamellipodial structures [38,39]. Furthermore, EMT is accompanied by distinct changes in the lipid composition and membrane organization, leading to increased lipid fluidity [40,41]. Collectively, these alterations work in concert to enhance the structural plasticity and migratory capacity of cells. In addition to the increased motility, EMT frequently confers resistance to anti-tumor drugs, including paclitaxel [42,43,44]. Although EMT is essential for normal processes in the body, such as embryonic morphogenesis, wound healing, and tissue remodeling [33,45], cancer cells exploit this process to acquire motile and invasive properties that drive cancer progression.

Despite the importance of EMT in cancer progression, our current knowledge on how EMT influences the biological effects of nsPEFs is limited. To explore this point, we analyzed the responses to nsPEFs in human A549 cells undergoing EMT. By comparing with non-EMT counterparts, we found that EMT has a significant effect on cellular susceptibility to nsPEFs. Our findings uncover a novel feature of the nsPEF effect and further strengthen the rationale for considering nsPEFs as a unique and promising approach to cancer therapy.

## 2. Results

### 2.1. Induction of EMT in A549 Cells by TGF-β1

**Figure 1:**
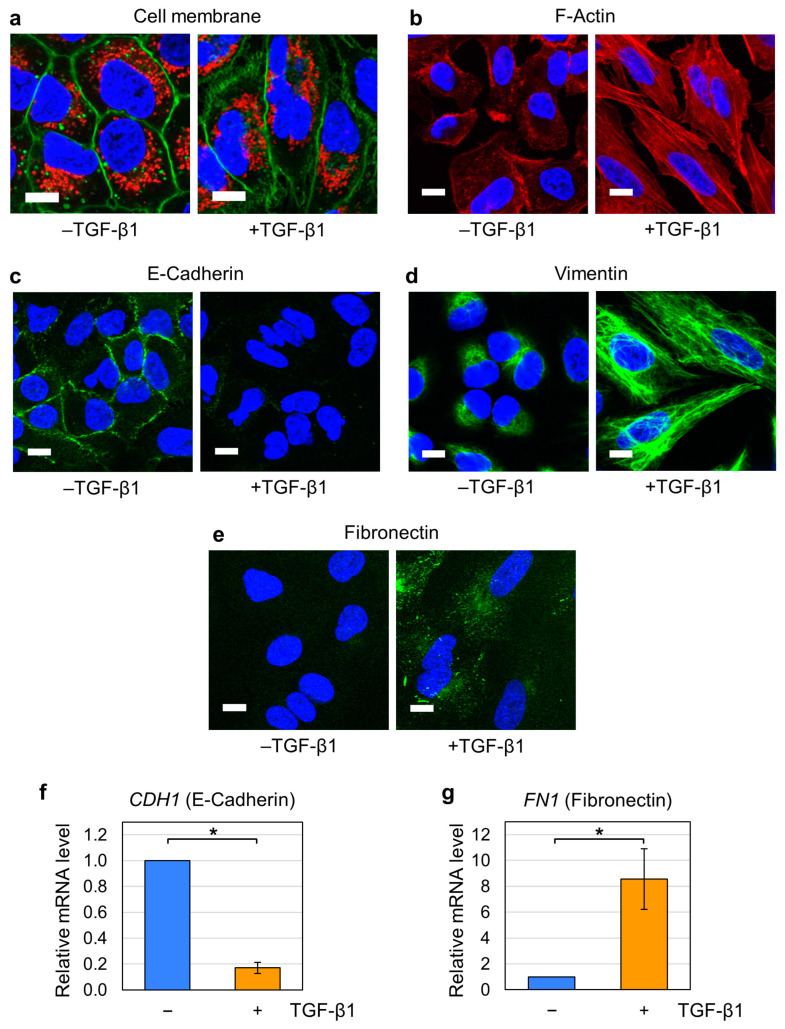
Induction of EMT in A549 cells. A549 cells were treated with or without 5 ng/mL TGF-β1 for 48 h. (**a**) Fluorescent co-staining of living cells for the cell membrane (green), the nucleus (blue), and mitochondria (red). Bar: 10 µm. (**b**) Fluorescent staining of fixed cells for F-actin (red) and DNA (blue). Bar: 10 µm. (**c,d**) Immunostaining for E-cadherin (**c**), vimentin (**d**), and fibronectin (**e**). DNA was costained (blue). Bar: 10 µm. (**f**,**g**) Quantitative RT-PCR for *CDH1* mRNA (**f**) and *FN1* mRNA (**g**). Average values with SD are shown (*n* = 5). *: Statistically significant (*p* < 0.01).

A549 cells are widely utilized as a model system for EMT induction in vitro, and TGF-β1 treatment is a well-established method for this purpose [46,47,48]. In this study, we treated A549 cells with 5 ng/mL TGF-β1 for 48 h to induce EMT. Because EMT is associated with morphological and molecular changes [35,36,47,49], we confirmed EMT induction by several analytical approaches. A list of reagents is shown in the Supplementary Information. First, we examined the morphologies of the cell membrane and nucleus by fluorescent staining. As shown in Figure 1a, A549 cells without TGF-β1 treatment displayed a typical epithelial morphology, characterized by polygonal shapes with well-defined membrane outlines (green) and round nuclei (blue). In contrast, TGF-β1-treated cells showed mesenchymal features: a spindle-like appearance with finely undulating contours (green) and nuclear deformation with indentation (blue) (Figure 1a, right). These morphological alterations collectively confirm the induction of EMT in TGF-β1-treated A549 cells.

Next, we analyzed the intracellular distribution of filamentous actin (F-actin). Cells were fixed and stained using phalloidin, which specifically binds to F-actin. In the absence of TGF-β1 treatment, intense red fluorescence was observed along the cell periphery, while cytoplasmic red staining was weak (Figure 1b, left), indicating that most F-actin was organized as a cortical actin network. In contrast, TGF-β1–treated cells exhibited prominent red fluorescence of cytoplasmic stress fibers (Figure 1b, right). These observations demonstrate that TGF-β1 treatment induced F-actin reorganization characteristic of EMT [38,39].

Finally, we analyzed the expression of membrane proteins associated with EMT. It is well documented that E-cadherin is present in epithelial cells and is downregulated during EMT [33,36,37]. Conversely, vimentin and fibronectin are known to be low in epithelial cells and upregulated during EMT [33,37]. We performed immunofluorescence analysis for these proteins. Green fluorescence for E-cadherin was detected in the periphery in untreated cells (Figure 1c, left) and was markedly reduced after TGF-β1 treatment (Figure 1c, right). We next immunostained for vimentin and observed characteristic intracellular distribution: perinuclear localization without TGF-β1 treatment (Figure 1d, left) and filamentous extension after TGF-β1 treatment (Figure 1d, right). As shown in Figure 1e, fibronectin was barely detectable in untreated cells and strongly induced following TGF-β1 treatment. We then quantified the expression of mRNAs for E-cadherin and fibronectin by quantitative RT-PCR. As shown in Figure 1e,f, E-cadherin expression decreased, and fibronectin expression increased upon EMT induction. These changes in the membrane protein expression are consistent with EMT induction. Collectively, these results confirm that A549 cells underwent phenotypic conversions characteristic of EMT under our experimental conditions. Hereafter, A549 cells with or without TGF-β1 treatment are referred to as EMT cells and non-EMT cells, respectively.

### 2.2. Increased Susceptibility of EMT Cells to nsPEFs

**Figure 2:**
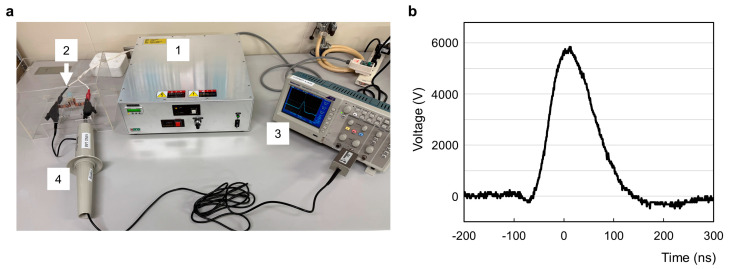
Generation of nsPEFs. (**a**) Experimental setup for the exposure of cell suspension to nsPEFs. 1: nsPEF generator. 2: Cuvette with a pair of 4 mm–gapped aluminum electrodes. 3: Oscilloscope. 4: High-voltage probe. (**b**) Representative wave form of nsPEFs. An electric pulse of 6000 V in a 4–mm gapped electrode is equivalent to electric field of 15 kV/cm. Pulse width at half maximum was estimated to be approximately 100 ns.

We next tested the effects of nsPEFs on cell viability using EMT and non-EMT cells. Figure 2a shows the setup for generating nsPEFs. We used 15 kV/cm nsPEFs (6000 V in a 4 mm-gapped cuvette), and the pulse width at half maximum was estimated to be approximately 100 ns (Figure 2b).

**Figure 3:**
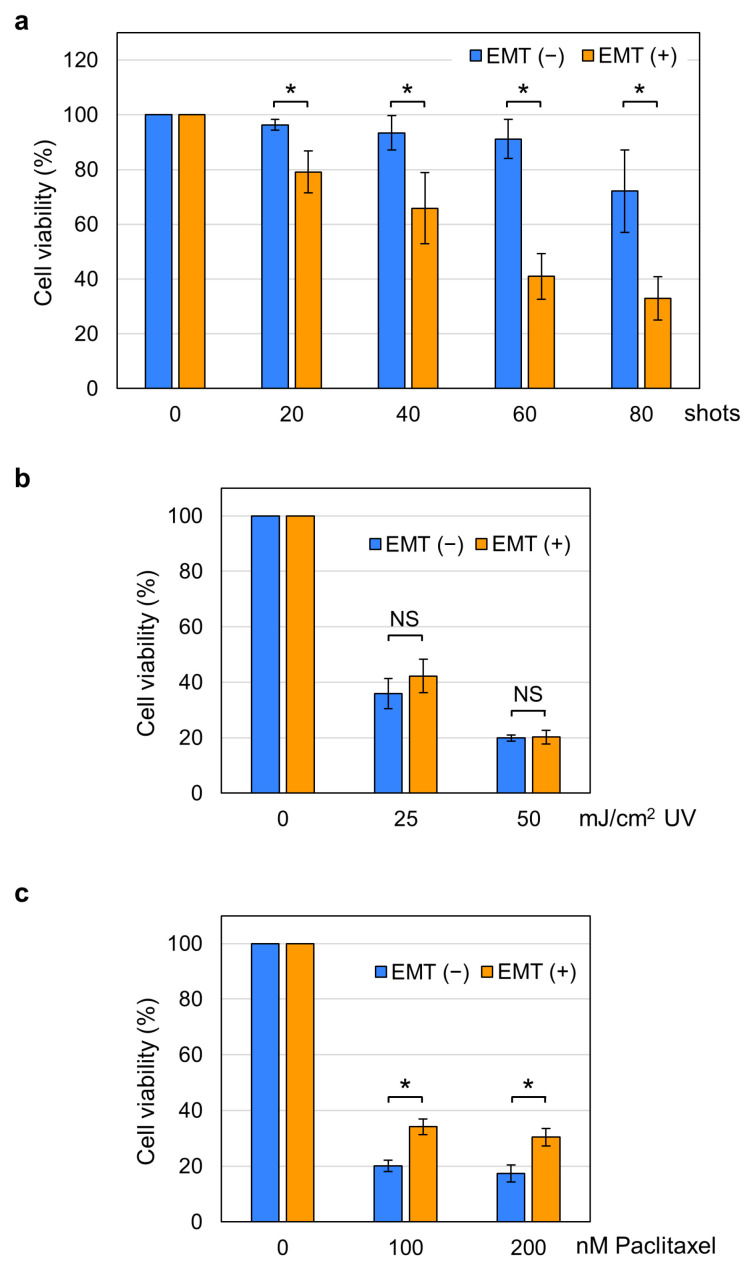
Effects of nsPEFs, UV, and paclitaxel on the viability of EMT and non-EMT cells. (**a**) Effects of nsPEFs. Either EMT or non-EMT cells were exposed to the indicated shot numbers of 15 kV/cm nsPEFs, and viability was assessed at 24 h. *: Statistically significant (*p* < 0.01, *n* = 5). (**b**) Effects of UV irradiation. Cells were irradiated with either 0, 25, or 50 mJ/cm^2^ 312 nm UV rays, and viability was measured at 24 h. NS: Not significant (*p* > 0.01, *n* = 5). (**c**) Effects of paclitaxel. Cells were treated with either 0, 100, or 200 nM paclitaxel for 48 h. *: Statistically significant (*p* < 0.01, *n* = 5).

We applied 20–80 shots of 15 kV/cm nsPEFs to EMT and non-EMT cells and measured cell viability 24 h post-treatment. As shown in Figure 3a, we observed significant differences in nsPEF susceptibility between EMT and non-EMT cells. Non-EMT cells were relatively resistant to nsPEFs, showing relatively slight decreases in cell viability. In contrast, the viability of EMT cells was markedly reduced by nsPEFs (Figure 3a). To examine whether EMT cells have elevated susceptibility to other cellular insults, we next tested additional physical and chemical treatments, namely UV irradiation and paclitaxel. As shown in Figure 3, the effect of UV irradiation was comparable between EMT and non-EMT cells (Figure 3b), and EMT cells were more resistant to paclitaxel than non-EMT cells (Figure 3c). These observations indicate that the higher susceptibility of EMT cells is specific to nsPEFs.

### 2.3. Increased Nanopore Formation by nsPEFs in EMT Cells

**Figure 4:**
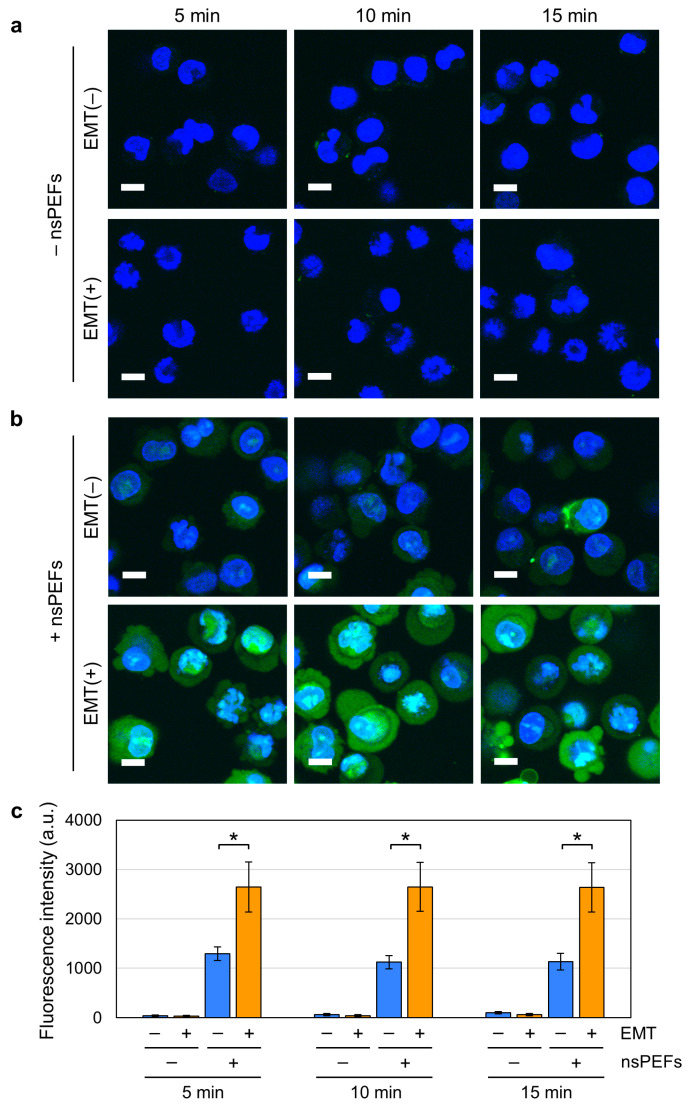
Nanopore formation by nsPEFs in EMT and non-EMT cells. (**a,b**) Representative images of A549 cells co-stained with YO-PRO-1 (green) and Hoechst 33342 (blue) without nsPEF exposure (**a**) or with 60 shots of 15 kV/cm nsPEFs (**b**). Bar: 10 µm. (**c**) Quantification of YO-PRO-1 fluorescence in cells. Fluorescence intensities of YO-PRO-1 in 30 cells were quantified at each time point in arbitrary units (a.u.). *: Statistically significant (*p* < 0.01, *n* = 5).

nsPEF exposure is well known to generate nanopores in the cell membrane, which can profoundly disturb cellular homeostasis [4,5,6,9,10]. Because EMT is accompanied by extensive remodeling of the cell membrane, we inferred that the vulnerability of the cell membrane might differ between EMT and non-EMT cells. To address this possibility, we next analyzed nsPEF-induced nanopore formation using YO-PRO-1, a green fluorescent dye that permeates nanopores but not intact membranes [4,6]. Cells were costained with Hoechst 33342, a membrane-permeant blue fluorescent dye for the visualization of nuclei. Without nsPEF exposure, we observed apparent blue staining of the nuclei and only faint green fluorescence, indicating that the cell membranes were largely intact in both EMT and non-EMT cells (Figure 4a). Next, we inspected nsPEF-exposed cells and observed green fluorescence in both EMT and non-EMT cells (Figure 4b). Notably, green fluorescence in EMT cells was much stronger than in non-EMT cells, indicating that more nanopores were generated in EMT cells (Figure 4b). For verification, we next quantified the green fluorescence and confirmed that it was significantly higher in nsPEF-exposed EMT cells than in non-EMT cells (Figure 4c). These results demonstrate that nsPEFs generate more nanopores in EMT cells than in non-EMT cells, suggesting that EMT increases membrane vulnerability to nsPEFs.

### 2.4. Induction of Non-Apoptotic Cell Death by nsPEFs in EMT Cells

**Figure 5:**
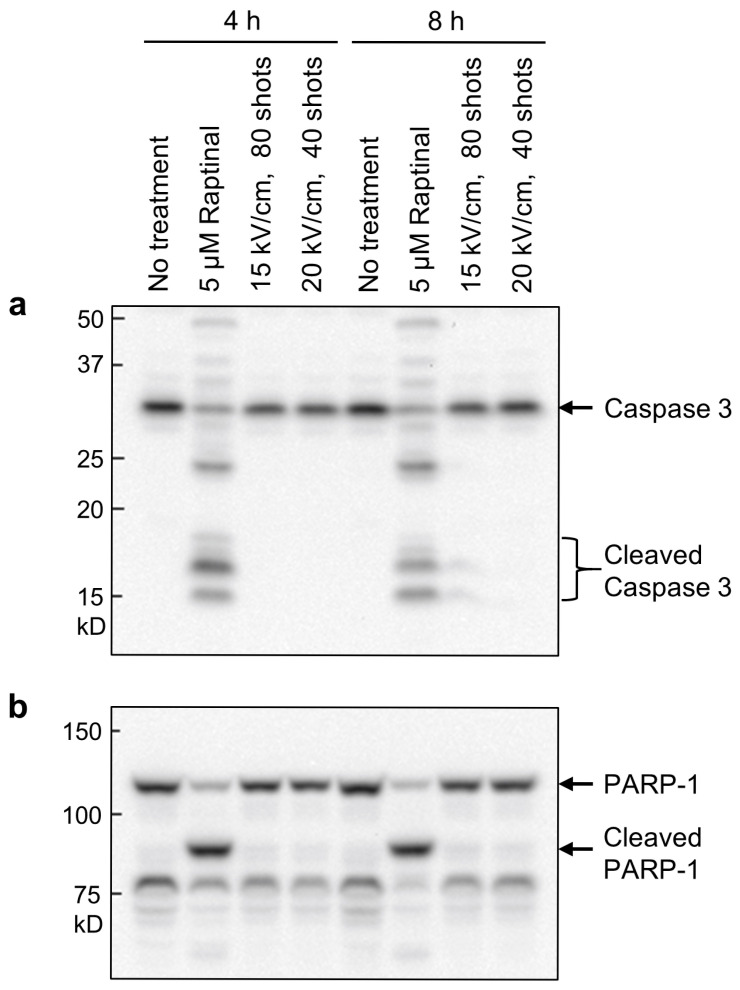
Western blot analysis of apoptosis-induced proteolytic cleavage. EMT cells were treated with either nsPEF exposure or Raptinal and incubated for 4 or 8 h. Caspase 3 (**a**) and PARP-1 (**b**) were analyzed by Western blotting.

Previous studies have demonstrated that nsPEFs induce either apoptotic or non-apoptotic cell death [18,19,20,21,22]. To further investigate the effects of nsPEFs on EMT cells, we analyzed the mode of cell death in nsPEF-exposed cells. First, we examined apoptosis-associated proteolysis of caspase-3 (Figure 5a) and PARP-1 (Figure 5b) in EMT cells by Western blotting. Among various apoptotic changes, the cleaved forms of these proteins are considered definitive markers of apoptosis, because their presence indicates that the cell has passed through the point of no return in the apoptotic cascade [50,51,52]. As a positive control for apoptosis induction, EMT cells were treated with Raptinal, a potent apoptosis-inducing compound [53]. As shown in Figure 5, both caspase-3 and PARP-1 remained uncleaved in nsPEF-exposed cells, whereas their cleaved forms were readily detectable in Raptinal-treated cells. The absence of these protein cleavages indicates that nsPEF-exposed EMT cells undergo non-apoptotic cell death under our experimental conditions.

**Figure 6:**
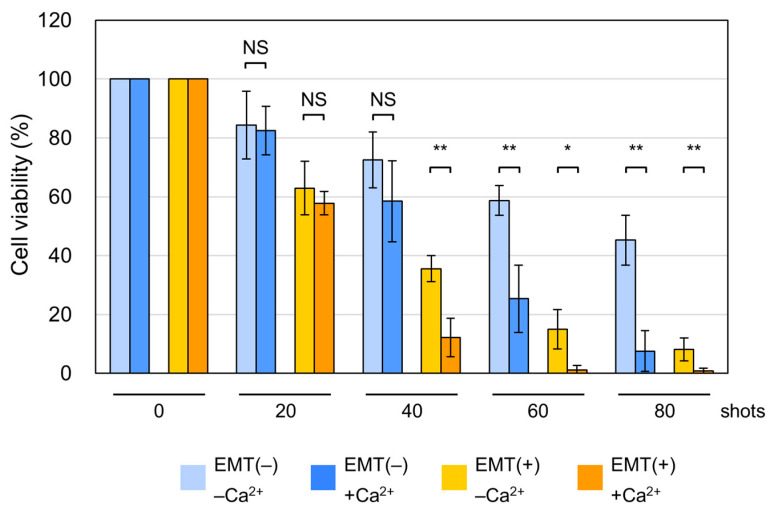
Effects of Ca^2+^ on the cell viability after nsPEF exposure. EMT and non-EMT cells were suspended in either Ca^2+^-free or Ca^2+^-containing DMEM and exposed to the indicated shot numbers of 15 kV/cm nsPEFs. Cell viability was measured at 24 h after nsPEF exposure. Average values with SD are shown (*n* = 5). NS: Not significant (*p* > 0.01). **: Statistically significant (*p* < 0.01). *: Statistically significant (0.01 < *p* < 0.05).

Previous studies have shown that the presence of extracellular Ca^2+^ affects the cellular susceptibility to nsPEFs during non-apoptotic cell death [21,54]. We thus investigated the effect of extracellular Ca^2+^ on the viability of EMT and non-EMT cells exposed to nsPEFs. We prepared a Ca^2+^-free medium (−Ca^2+^ medium) by mixing Ca^2+^-free DMEM and dialyzed Ca^2+^-free serum. Ca^2+^-containing medium (+Ca^2+^ medium) was prepared by adding CaCl_2_ to −Ca^2+^ medium at a physiological concentration (1.8 mM). Cell suspension was prepared in either −Ca^2+^ or +Ca^2+^ medium and used for the viability assay. As shown in Figure 6, the presence of Ca^2+^ resulted in lower viability at higher numbers of nsPEF shots in both EMT and non-EMT cells: in EMT cells, the difference in cell viability between −Ca^2+^ or +Ca^2+^ conditions was evident at 40, 60, and 80 shots, but not 20 shots of nsPEFs (Figure 6). In non-EMT cells, the presence of Ca^2+^ caused greater susceptibility to nsPEFs at 60 and 80 shots, but the differences were not statistically significant at lower shots (20 and 40 shots) (Figure 6). These results indicate that Ca^2+^ enhances the susceptibility of EMT and non-EMT cells to high numbers of nsPEF shots.

## 3. Discussion

nsPEFs are regarded as a potential modality for cancer therapy because of their ability to induce cell death in vitro and to effectively ablate tumors in animal models [1,2,3]. EMT is a critical step in cancer progression, as it endows cancer cells with malignant traits associated with metastasis and invasiveness [33,34,35]. Despite the biological and medical importance of EMT, however, little is known about how EMT affects the effect of nsPEFs. We thus investigated the responses of EMT-induced and non-induced A549 cells to nsPEF exposure. We found that EMT cells were more susceptible to nsPEFs than non-EMT cells (Figure 3a). The higher susceptibility of EMT cells was characteristic of nsPEFs, as it was not observable when the cells were UV-irradiated or treated with paclitaxel (Figure 3b,c). These findings reveal a novel feature of nsPEFs in targeting EMT cells, providing further support for their potential effectiveness in cancer therapy. In addition to the therapeutic implications, our study provides an experimental demonstration that two distinct physiological states, namely EMT-induced and uninduced, within the same genetic background can confer differential susceptibility to nsPEFs.

We observed higher susceptibility (Figure 3a) and increased YO-PRO-1 incorporation (Figure 4) in nsPEF-exposed EMT cells. Because various alterations in proteins and lipids of the cell membrane occur during EMT [35,36,37,38,39,40,41], we infer that these changes may cumulatively affect membrane vulnerability to nsPEFs, leading to the observed higher susceptibility for several reasons. First, EMT cells are characterized by the downregulation of epithelial proteins on the membrane (e.g., E-cadherin, Figure 1c,e) and upregulation of mesenchymal proteins (e.g., fibronectin, Figure 1d,f) [36,37], both of which may be associated with the decreased membrane resilience against nsPEF exposure.

Second, the reduction in the actin cortex during EMT may increase the fragility of the cell membrane to nsPEFs. The actin cortex is a dense layer of F-actin and anchored to the cell membrane via membrane proteins, such as E-cadherin. As E-cadherin expression is downregulated during EMT, actin molecules are redirected from the cortex to assemble into cytoplasmic stress fibers [38,39]. The reduction in the actin cortex during EMT is considered to be a trade-off between migratory capacity and mechanical strength [55,56]. Thus, the higher susceptibility to nsPEFs may be ascribed to the membrane vulnerability arising from the decreased actin cortex.

Next, the lipid profiles of the cell membrane may affect the vulnerability of the membrane to nsPEFs. EMT is accompanied by changes in membrane lipid profiles, which increase membrane fluidity and cell motility at the expense of reduced mechanical strength [40,41]. Because previous studies have demonstrated that the composition of membrane lipids influences nanopore formation by nsPEFs [57], altered membrane lipid profiles during EMT may increase membrane vulnerability and consequently enhance cell susceptibility to nsPEFs.

Taken together, because EMT involves substantial alterations in both proteins and lipids that promote motility and invasiveness, we speculate that the observed higher membrane vulnerability and cell susceptibility to nsPEFs may be attributed to EMT-associated structural remodeling. In future investigations, the possible link between EMT-associated membrane remodeling and increased nsPEF susceptibility should be directly verified using biophysical methods, such as atomic force microscopy. Although the observed increased nsPEF susceptibility is consistent with known changes in membrane properties during EMT, the biophysical analysis of the membrane will establish a mechanistic basis for this relationship.

Previous studies have reported that nsPEFs induce non-apoptotic cell death in certain cell types [19,20,21], and the present work similarly demonstrates non-apoptotic cell death in EMT cells (Figure 5). At present, nsPEF-induced non-apoptotic death appears to deviate from well-characterized pathways such as necroptosis and ferroptosis. Consistent with earlier studies [11,12,13], our data indicate the involvement of Ca^2+^ in the cytotoxicity of nsPEFs (Figure 6). Sustained Ca^2+^ elevation is known to have detrimental effects on phosphate-containing molecules, including ATP and nucleic acids, because Ca^2+^ binds tightly to phosphate groups and reduces the solubility and conformational flexibility of these molecules, ultimately leading to diverse cellular defects [58]. In line with this, Ca^2+^-dependent nsPEFs cytotoxicity is frequently accompanied by decreased mitochondrial activity and reduced intracellular ATP levels [11,12,13]. Because a previous study has reported the induction of apoptosis in A549 cells by nsPEFs with the participation of reticular Ca^2+^ [59], it can be assumed that excessive influx of extracellular Ca^2+^ may cause necrotic cell death due to reduced cellular energy. Elucidating the molecular mechanisms by which nsPEF exposure triggers this atypical form of cell death should therefore be an important focus of future studies.

Despite demonstrating a previously unrecognized aspect of nsPEF cytotoxicity in EMT cells, the present study has several limitations that should be addressed to advance the therapeutic application of nsPEFs. First, in this study, nsPEF effects were evaluated in only one epithelial cell line, A549. Because EMT responses and nsPEF susceptibility can vary among cell types, additional validation in other EMT-competent models will be important. Future studies should examine additional epithelial cell lines with well-characterized EMT plasticity to determine whether the EMT-associated differences observed here can be generalized. Second, it should be determined whether nsPEFs can preferentially eliminate EMT subpopulations within heterogeneous tumors. This could be experimentally evaluated using mixed-cell spheroid models, in which distinct cell states are spatially organized. Such systems would enable the direct assessment of whether nsPEFs exhibit higher cytotoxicity to EMT cells compared to their non-EMT counterparts in a tumor. In conclusion, further investigation to address these points will provide a better understanding of how nsPEFs act on EMT and non-EMT cells and pave the way toward better therapeutic applications of nsPEFs.

## 4. Materials and Methods

### 4.1. Cell Culture and TGF-β1 Treatment

A549 cells were obtained from RIKEN BioResource Research Center (Wako, Saitama, Japan) and grown in RPMI1640 medium (FUJIFILM Wako Pure Chemical, Osaka, Japan) with 10% fetal bovine serum (FBS) (Corning, NY, USA) and penicillin/streptomycin (FUJIFILM Wako Pure Chemical). Our standard culture conditions were at 37 °C in a humidified atmosphere with 5% CO_2_.

TGF-β1 (PeproTech, Thermo Fisher Scientific, Waltham, MA, USA) was dissolved in 10 mM citric acid solution at pH 3 [60] that contained 1 mg/mL bovine serum albumin. For EMT induction, TGF-β1 was added to the culture medium at 5 ng/mL, and the cell culture was continued for 48 h.

### 4.2. Generation of nsPEFs

The nsPEF generator CUS8000V-10P was designed and manufactured by Suematsu Electronics (Kumamoto, Japan). A voltage waveform was monitored using a high-voltage probe (P6015A, Tektronix, Beaverton, OR, USA) and an oscilloscope (DPO4054, Tektronix).

### 4.3. Exposure of Cells to nsPEFs

Cells were detached by trypsinization and suspended in appropriate culture medium containing 10% FBS. Cells were pelleted by centrifugation and resuspended in fresh culture medium. An aliquot (400 µL) of cell suspension was added to an electro-poration cuvette with 4 mm gapped electrodes (Bio-Rad Laboratories, Hercules, CA, USA). Shots of 15 kV/cm nsPEFs were applied to the cuvette at 1-s intervals.

### 4.4. Quantitative RT-PCR

Extraction of total RNA was performed using RNAiso plus (Takara Bio, Shiga, Japan). RT-PCR was conducted using an iTaq Universal SYBR Green One-Step Kit (Bio-Rad Laboratories). The process of PCR was monitored using an MJ Mini Thermal Cycler equipped with a MiniOpticon Real-Time PCR system (Bio-Rad Laboratories). Average cycle thresholds were evaluated using CFX Manager Software (Version 1.6, Bio-Rad Laboratories) and normalized to the values of *GAPDH* mRNA. The Cq values of qPCR and gel images are shown in Supplementary Figure S1. Primer sequences used were:

*CDH1* (E-cadherin) forward, 5′-GTGGTTCAAGCTGCTGACCT-3′.

*CDH1* (E-cadherin) reverse, 5′-CTGACCCTTGTACGTGGTGG-3′.

*FN1* (fibronectin) forward, 5′-CCGGGACTCAATCCAAATGCC-3′.

*FN1* (fibronectin) reverse, 5′-TTCCAGGAACCCTGAACTGTAAGG-3′.

*GAPDH* forward, 5′-GCACCGTCAAGGCTGAGAAC-3′.

*GAPDH* reverse, 5′-TGGTGAAGACGCCAGTGGA-3′.

### 4.5. Fluorescence Microscopy

Fluorescence microscopy was conducted using an FV1200-IX83 microscope (Olympus, Tokyo, Japan), equipped with a stage-top incubator (Tokai Hit, Shizuoka, Japan) for live cell imaging. Fluorescent images were acquired using FLUOVIEW (Version 4.1, Olympus).

### 4.6. Fluorescent Staining of Living Cells

Cells were costained with PlasMem Bright Green for the cell membrane (200-fold dilution, Dojindo Laboratories, Kumamoto, Japan), Hoechst 33342 for the nuclei (1 µg/mL, Dojindo Laboratories), and MitoRed for mitochondria (0.2 µM, Dojindo Laboratories).

### 4.7. Fluorescent Staining of Fixed Cells

Cells were fixed with 4% paraformaldehyde and subsequently permeabilized with 0.4% Triton X-100 for 5 m. Cells were incubated with 1% bovine serum albumin in D-PBS for 15 m and then subjected to fluorescent staining of either F-actin, E-cadherin, vimentin, or fibronectin as follows. For F-actin staining, cells were reacted with phalloidin-iFluor 555 (Cayman Chemical, Ann Arbor, MI, USA) and mounted in a Vectashield mounting medium with DAPI (Vector Laboratories, Newark, CA, USA). For E-cadherin, vimentin, and fibronectin, cells were reacted with rabbit anti-E-cadherin antibody (24E10, Cell Signaling Technology, Danvers, MA, USA), rabbit anti-vimentin antibody (#5741, Cell Signaling Technology) and rabbit anti-fibronectin antibody (E5H6X, Cell Signaling Technology), respectively. Cells were subsequently incubated with anti-rabbit Alexa Fluor488 antibody (Thermo Fisher Scientific) and mounted in Vectashield with DAPI as described above.

### 4.8. YO-PRO-1 Staining

Cell suspension was prepared as described above by use of culture medium containing YO-PRO-1 (1 µM, Thermo Fisher Scientific) and Hoechst 33342 (1 µg/mL, Dojindo Laboratories). Cell suspension with fluorescent dyes was exposed to shots of nsPEFs, and fluorescent images were captured at 5, 10, and 15 m. Fluorescence intensities in individual cells were measured in arbitrary units using FLUOVIEW software (Version 4.1, Olympus).

### 4.9. Cell Viability Assay

Cells were detached and suspended in RPMI1640 with 10% FBS as described above. After nsPEF exposure, an aliquot of cell suspension was incubated at 37 °C for 24 h. Cell viability was examined using a Cell Counting Kit-8 (Dojindo Laboratories) and an MPR-A100 microplate reader (AsOne, Osaka, Japan). UV irradiation was performed using a 312 nm UV irradiator (BLX-312, Vilber Lourmat, France), and cell viabilities were analyzed at 24 h as above. When paclitaxel (FUJIFILM Wako Pure Chemical) was used for treatment, cell viability was measured at 48 h.

For the analysis of Ca^2+^ effects, RPMI1640 medium and regular FBS were replaced with Ca^2+^-free DMEM (Thermo Fisher Scientific) and dialyzed FBS (Biological Industries, Beit-Haemek, Israel), respectively. A Ca^2+^-containing counterpart was prepared by adding CaCl_2_ at 1.8 mM to Ca^2+^-free DMEM with dialyzed FBS. Notably, when DMEM with dialyzed FBS was used, the viability of nsPEF-exposed cells was lower than that in RPMI with regular FBS (compare Figure 3 and Figure 6). This reduction in the viability may be attributable to differences in the composition between RPMI/regular FBS and DMEM/dialyzed FBS.

### 4.10. Western Blot Analysis

Cell suspension in RPMI medium was exposed to nsPEFs as described above. Raptinal was obtained from Tokyo Chemical Industry (Tokyo, Japan) and used at 5 µM as a positive control for apoptosis induction. After incubation for 4 or 8 h, cells were lysed and used for Western blot analysis using anti-caspase 3 antibody (#9662, Cell Signaling Technology) and anti-PARP-1 antibody (46D11, Cell Signaling Technology). A protein of interest was detected by a chemiluminescence method as described previously [15].

### 4.11. Statistical Analysis

The Mann–Whitney U test was performed for statistical analysis using R (Version 4.5.1, R Core Team, 2025, Auckland, New Zealand, https://cran.r-project.org/, accessed on 3 November 2025) and RStudio (Version 2025.09.2, Build 418; Posit Software, PBC, Boston, MA, USA, https://posit.co/download/rstudio-desktop/, accessed on 3 November 2025).

## Data Availability

The original contributions presented in this study are included in the article. Further inquiries can be directed to the corresponding author.

## Supplementary Materials

The following supporting information can be downloaded at: https://www.mdpi.com/article/10.3390/ijms262311360/s1.

## Abbreviations

The following abbreviations are used in this manuscript:

nsPEFs	Nanosecond pulsed electric fields
EMT	Epithelial–mesenchymal transition
