# Supplementary material for: Epithelial–Mesenchymal Transition Increases the Susceptibility of Human A549 Cells to Nanosecond Pulsed Electric Fields
Source: Int J Mol Sci. 2025 Nov 24;26(23):11360. doi: 10.3390/ijms262311360 (PMC12692578; doi:10.3390/ijms262311360)
Supplement: Supplementary file 1 [file ijms-26-11360-s001.zip › ijms-3934868-supplementary.pdf]

Supplementary Information

Epithelial-Mesenchymal Transition Increases the Susceptibility of Human A549 Cells to Nanosecond Pulsed Electric Fields

Manato Mitsui [1], Keiko Morotomi-Yano [2] and Ken-ichi Yano [1,2]

1. Faculty of Advanced Science and Technology, Kumamoto University, Kumamoto 860-8555, Japan
2. Institute of Industrial Nanomaterials, Kumamoto University, Kumamoto 860-8555, Japan

List of Reagents Used for the Evaluation of EMT Induction in Figure 1a - 1d.

Figure 1a: Living cells (unfixed cells)

Target	Reagent for detection	Color
Cell membrane	PlasMem Bright Green	Green
Mitochondria	MitoRed	Red
DNA	DAPI	Blue

Figure 1b: Fixed cells

Target	Reagent for detection	Color
F-actin	Phalloidin-iFluor555	Red
DNA	DAPI	Blue

Figure 1c: Fixed cells

Target	Reagent for detection	Color
E-Cadherin	rabbit anti-E-cadherin antibody<br>+ anti-rabbit Alexa Fluor488 antibody	Green
DNA	DAPI	Blue

Figure 1d: Fixed cells

Target	Reagent for detection	Color
Fibronectin	rabbit anti-fibronectin antibody<br>+ anti-rabbit Alexa Fluor488 antibody	Green
DNA	DAPI	Blue

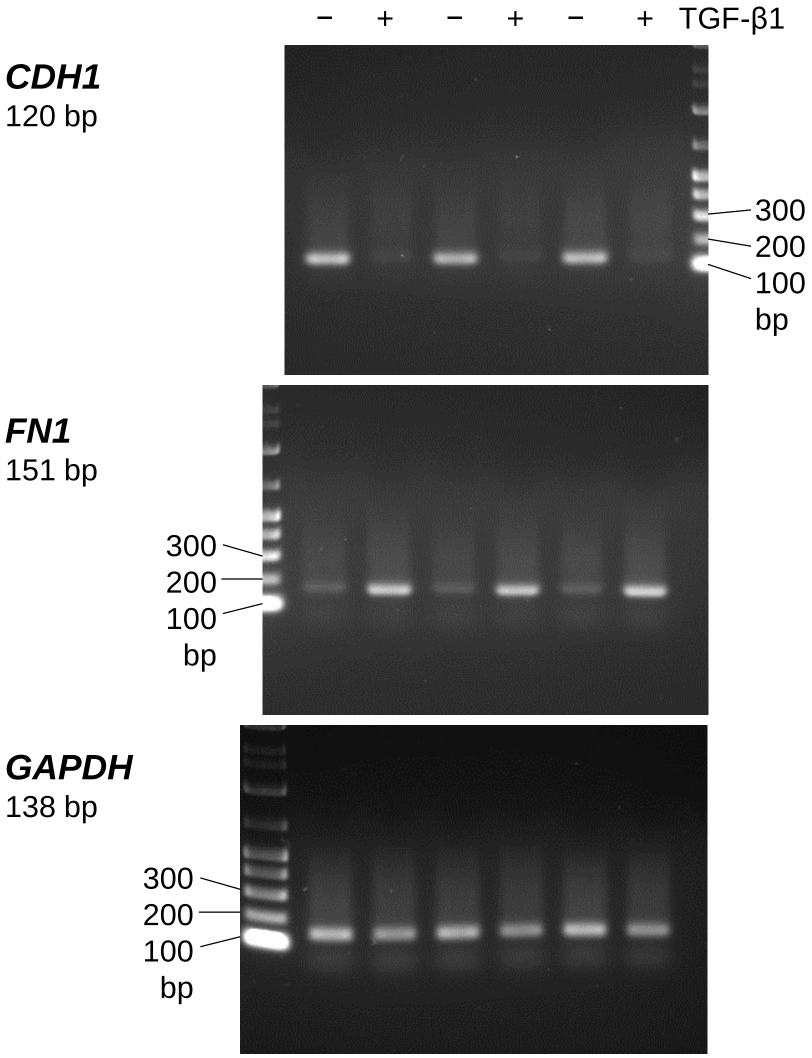

Cq Values

Target	Sample		Cq
CDH1	1	-	21.08
CDH1	2	-	20.95
CDH1	3	-	20.51
CDH1	4	+ EMT	24.36
CDH1	5	+ EMT	23.36
CDH1	6	+ EMT	24.1

Target	Sample		Cq
FN1	1	-	24.34
FN1	2	-	24.58
FN1	3	-	24.39
FN1	4	+ EMT	21.61
FN1	5	+ EMT	21.48
FN1	6	+ EMT	21.44

Target	Sample		Cq
GAPDH	1	-	17.04
GAPDH	2	-	16.92
GAPDH	3	-	16.96
GAPDH	4	+ EMT	17.27
GAPDH	5	+ EMT	17.06
GAPDH	6	+ EMT	17.59

Supplementary Figure S1. Results of RT-qPCR.
Reverse transcription followed by qPCR was performed as described in the Methods section. Results of three independent experiments are shown.
